# Supplementary material for: Role of ACSBG1 in Brain Lipid Metabolism and X-Linked Adrenoleukodystrophy Pathogenesis: Insights from a Knockout Mouse Model
Source: Cells. 2024 Oct 12;13(20):1687. doi: 10.3390/cells13201687 (PMC11506745; doi:10.3390/cells13201687)
Supplement: Supplementary file 1 [file cells-13-01687-s001.zip › cells-3201759-supplementary.pdf]

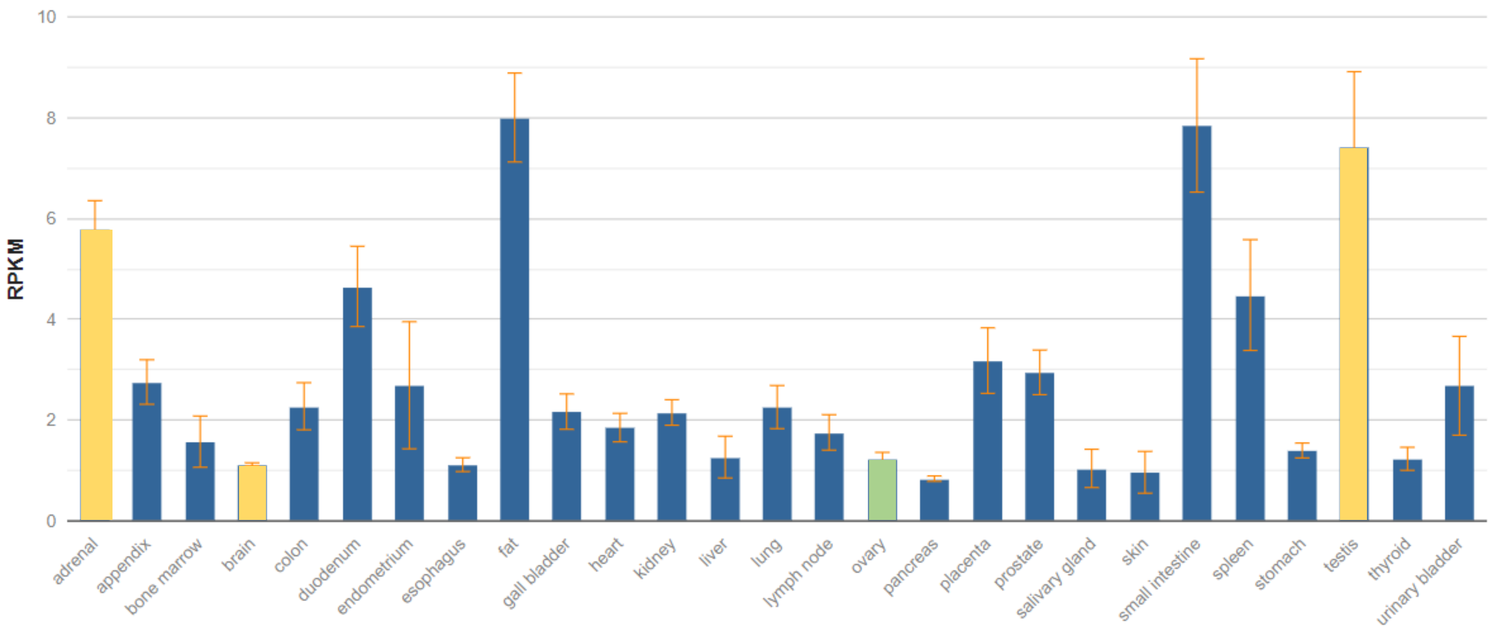

Figure S1. Relative expression of ABCD1 mRNA in tissues. RNAseq data for estimation of ABCD1 expression in human tissues [18] was obtained by first querying the National Center for Biotechnology Information (NCBI) gene database for homo sapiens ABCD1. Selecting ñexpressionò from the options menu generated the plot shown in this figure. Results are expressed as RPKM (Reads Per Kilobase per Million mapped reads). Tissues highlighted in yellow are tissues with pathology in XALD. These tissues, plus ovary (highlighted in green), express ACSBG1.

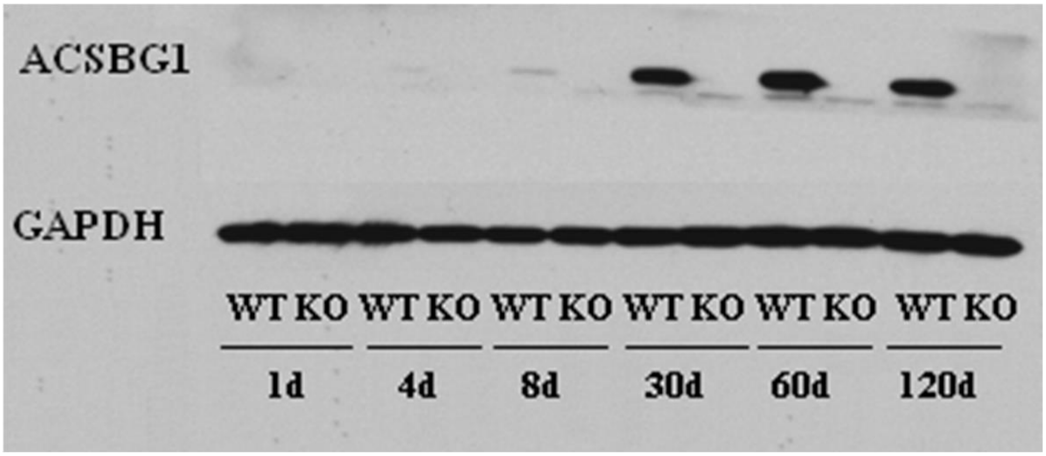

Figure S2. ACSBG1 protein expression in developing mouse cerebellum. Samples of cerebellum from w.t. or KO mice of increasing age were subjected to Western blotting as described in Methods. A representative blot is shown. ACSBG1 is detected as a ~70 kDa band. GAPDH was used as a loading control.

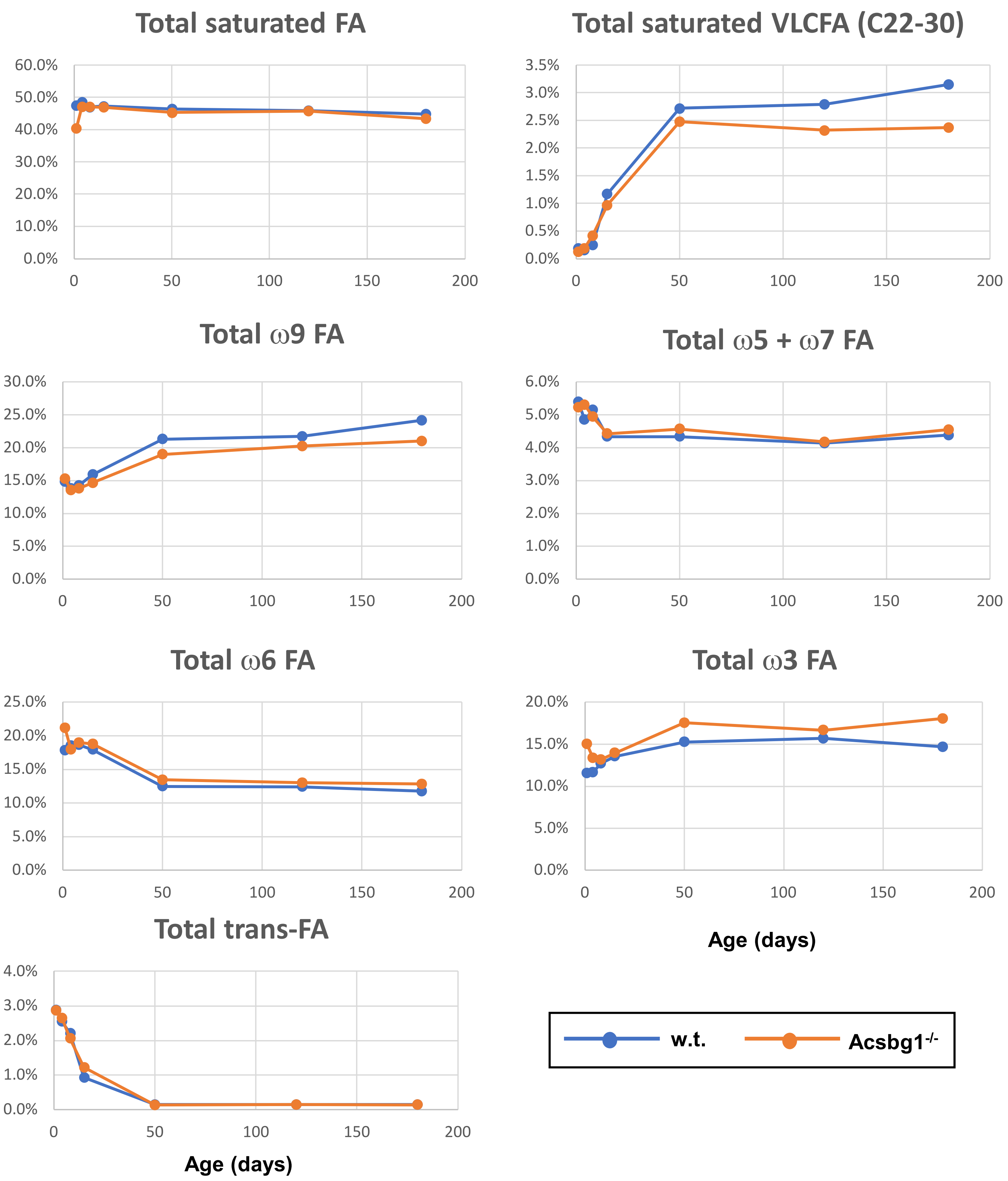

Figure S3. Change with age in different classes of cerebellar FA in w.t. and Acsbg-/- mice. Lipids were extracted from cerebellum and FA levels quantitated as described in the legend to Table 4. Levels of FA in different classes are shown as a percentage of total FA.
